# Episodic Reasoning for Vision-Based Human Action Recognition

**DOI:** 10.1155/2014/270171

**Published:** 2014-05-14

**Authors:** Maria J. Santofimia, Jesus Martinez-del-Rincon, Jean-Christophe Nebel

**Affiliations:** ^1^Computer Architecture and Network Group, School of Computer Science, University of Castilla-La Mancha, 13072 Ciudad Real, Spain; ^2^The Institute of Electronics, Communications and Information Technology (ECIT), Queens University of Belfast, Belfast BT3 9DT, UK; ^3^Digital Imaging Research Centre, Kingston University, London KT1 2EE, UK

## Abstract

Smart Spaces, Ambient Intelligence, and Ambient Assisted Living are environmental paradigms that strongly depend on their capability to recognize human actions. While most solutions rest on sensor value interpretations and video analysis applications, few have realized the importance of incorporating common-sense capabilities to support the recognition process. Unfortunately, human action recognition cannot be successfully accomplished by only analyzing body postures. On the contrary, this task should be supported by profound knowledge of human agency nature and its tight connection to the reasons and motivations that explain it. The combination of this knowledge and the knowledge about how the world works is essential for recognizing and understanding human actions without committing common-senseless mistakes. This work demonstrates the impact that episodic reasoning has in improving the accuracy of a computer vision system for human action recognition. This work also presents formalization, implementation, and evaluation details of the knowledge model that supports the episodic reasoning.

## 1. Introduction

Recognizing human actions is an essential requirement for fulfilling the vision of Smart Spaces, Ambient Intelligence, or Ambient Assisted Living. These paradigms envision environments in which electronic devices, merged with the background, operate as sensors retrieving environmental information. Among all different types of sensors, video cameras are extremely powerful devices because of the great amount of contextual information that they are capable of capturing. However, despite human's ability to understand effortlessly video sequences through observation, computer vision systems still have work to do in this regard.

Automatic video understanding is a delicate task that yet remains an unresolved topic [1]. Among all the challenges involved in video understanding, this paper focuses on human action recognition since this is an enabling key for Smart Spaces applications. Applications that depend on the identification of certain behavior require the ability to recognize actions. For example, kicking and punching are two actions that suggest an ongoing fight. In this sense, having the ability to recognize the sequence of actions that define an undesirable behavior can be used to trigger a security alarm.

Obviously, several challenges arise when dealing with human action recognition. In addition to the inherent difficulty of recognizing different people's body postures performing the same action [2], different actions may involve similar or identical poses. Moreover, images recorded within a real environment are not always captured from the best perspective or angle, which makes it impossible to retrieve poses consistently [3].

Fortunately, the human ability to recognize actions does not only rely on visual analysis of human body postures but also requires additional sources of information such as context, knowledge about actor intentions, or knowledge about how the world works normally referred to as common sense. This type of information helps people to recognize, among several similar actions, the one that is the most consistent with knowledge that person holds about previous experiences. For example, consider the actions of waving and throwing something overhead. They can be performed quite in the same way. However, if it is known beforehand that the actor is not holding anything that could be thrown away, waving is the most likely action being performed.

However, this human ability is far more sophisticated than just a simple condition matching process. People have also the capacity to hypothesize about different situations or episodes, project effects of actions based on previous experiences, wait for following actions to explain a previously nonunderstood action, or even ignore the occurrence of a certain action that cannot be recognized without interfering in the interpretation and understanding of the situation. Human's episodic memory is what enables us to model, represent, and reason about events, actions, preconditions, and consequences [4].

An episode is considered here to extend along time and involve a sequence of events and actions, presided by an undergoing plan. In this sense, a single action, such as walking, should be seen as part of a higher level action, activity, or episode such as approaching an object to pick it up. Single actions can take place on isolation but, for understanding purposes, it is essential to distinguish when they are being part of a more complex activity or episode. Note that the words activity and episode are used instinctively along this document.

Consequently, a successful action recognition system, inspired by the human one, should entail two different perspectives, that is, visual analysis and episodic reasoning. This work extends the work in [5] in which an off-the-shelf computer vision system is combined with a heuristic system for human action recognition. This work improves the heuristic system by providing a computational implementation of the human episodic memory paradigm to support episode modeling, representation, and reasoning. Different mental models intervene in episodic reasoning and this work proposes use of three of them:* beliefs, expectations, and estimations*. These mental models hold different implications, such as the fact that a belief is true in the context of the person that holds that idea although it may not be true in the real world context. These implications have to be captured in order to successfully implement an episodic reasoning approach. A preliminary attempt to present this formulation was introduced in [6]. Furthermore, usage of these mental models is formalized by means of a semantic model and validated by, first, translating them into a software implementation and, second, assessing the commonsensicality level of the resulting system.

The following sections describe how these endeavors can be articulated based on the implementation of philosophical theories about knowledge and human behavior. More particularly, Section 2 presents some of the most relevant works going in the same direction as the one presented here.  Section 3 discusses the relevance that common sense has in achieving system with intelligent capabilities.  Section 4 proposes and formalizes a knowledge model for video-based human action recognition.  Section 5 describes how the formal theories supporting this work can be implemented into concrete procedures that can be computationally run.  Section 6 validates the working hypothesis motivating this work by assessing the proposed system performance, from both the computer vision perspective and the human cognition one. Finally, Section 7 presents the most relevant conclusions drawn from the work described here.

## 2. Previous Work

Different approaches have been devised to tackle the problem of human action recognition from the computer vision perspective, such as [7–10]. Mainly, video-based action recognition algorithms rely on learning from examples and machine learning techniques such as HMM [11], dimensionality reduction [12–14], or Bag of Words [9]. Since these approaches do not include any reasoning capability, their efficiency relies completely on the training and its coverage of all actions present in a given scenario. Unfortunately, all those action recognition experiments are conducted with videos that are not representative of real life data, as it is demonstrated by the poor performance obtained on videos captured in uncontrolled environments [9, 15]. It has been concluded that none of existing techniques based solely on computer vision and machine learning is currently suitable for real video surveillance applications [1].

However, few works combine video-based strategies with anthropological aspects or knowledge about human and social behavior [16], despite these essential elements being of human behavior [17]. According to [18] human behavior is enabled by six different types of mechanisms: instinctive reactions, learned reactions, deliberative thinking, reflective thinking, self-reflective thinking, and self-conscious reflection. These mechanisms should be therefore considered as an inherent part of any system intended to understand human behavior, independent of the dimension in which it is expressed, thinking, acting, or talking, for example. On the contrary, the approach that followed from the human action recognition perspective consists in rather equipping systems with the minimum amount of information required to solve the problem.

Enabling computational systems with these mechanisms is not an accessory demand, but, on the contrary, it is becoming more and more essential as new paradigms depend on showing rational behavior. In this sense, human activity recognition is becoming a hot topic due to the key role it plays in fields of knowledge such as Ambient Assisted Living (AAL) [19] or Ambient Intelligence (AmI) [20]. In fact, as stated in [21], activity recognition is one of the main challenges faced by AAL [22]. Provided that human activity recognition is a task that can be framed in very different fields of knowledge, it is important to state here that this work focuses on achieving video-based human action recognition as an enabling key for AAL spaces. These spaces are characterized by showing skills for supervising, helping, and assisting the elderly in their daily life. These skills need to therefore be grounded in cognitive and understanding capabilities.

This reference to cognitive and understanding capabilities basically alludes to computational mechanisms for interpreting the facts provided by sensors and video devices deployed in an AAL space. The events captured by environmental sensors are interpreted as signal describing an ongoing episode in a well-known context. Modeling the knowledge and information gathered from this type of scenarios has also been a major topic of discussion. In this sense, the World Wide Web Consortium (W3C), aware of that shortage, provides a standardized and formal model of the environment [23]. Despite this attempt to standardize the conceptual entities that should be part of the model, this ontology fails to provide the means to model ongoing episodes or situations, and, for that reason, the work presented here has adopted the model proposed by McCarthy and Hayes [24]. The* situation* concept proposed by McCarthy models world episodes as changes result of actions and events taking place in it. This work has therefore adopted this approach by describing actions and events in terms of a set of statements describing the world before the action takes place and afterward.

Setting aside the formality employed for knowledge modeling, next issue to be considered is the employed mechanism for undertaking human action recognition. Despite the fact that there is not a unique standard procedure for action recognition in AAL, some of the most common approaches are rule-based [21, 25, 26], statistical [27] or learning, both in supervised and unsupervised modes [28, 29]. However, due to the background of this paper, special attention is paid to those approaches based on video, like the one presented here. The work in [30] employs human silhouettes linked by connectors, in such a way that different postures are represented by means of different silhouettes and connections. The work in [31] proposes decomposing human actions into subtasks, such that the recognition process is accomplished in different stages. The work in [32], despite not being specifically devoted to a video-based solution, refers to sensors in general so it can be easily extrapolated to video-based systems. It consists in applying statistical modeling of sensor behavior to learn behavioral patterns that can be used for security and care systems. The work in [33] extends the previous work to consider additional approaches for not only monitoring systems but also making special emphasis on the behavior modeling task. However, these types of systems, mainly characterized by their rigidness, fail to deal with unexpected or unforeseen situations. For that reason, more elaborated reasoning mechanisms are required to deal with action recognition in open spaces. By open spaces we refer here to those environments in which interactions and events are coming from different sources at unexpected times.

The task of modeling human behavior has been tackled in this work from the perspective of common sense. Some activities have already been undertaken in this regard, although from the perspective of indoor mobile robots [34, 35]. Due to the great effort involved in collecting knowledge about the everyday world, the most commonly employed approach consists in resorting to existing systems. There are not many systems dedicated to collect and manage common-sense knowledge. In fact, the most famous ones are OpenMind (http://commons.media.mit.edu/en/), Cyc or OpenCyc (http://www.opencyc.org/), and Scone (http://www.cs.cmu.edu/~sef/scone/). The first system simply provides knowledge-based capabilities, lacking of an inference and reasoning engine, similarly, although OpenCyc holds these mechanisms, it only provides limited capability in comparison with the commercial project Cyc. Finally, Scone is an open-source system that provides efficient mechanisms for supporting common-sense reasoning and knowledge modeling operations [4, 36]. The characteristic that makes Scone the most suitable choice when it comes to episodic reasoning is its capability to deal with multiple contexts at the same time. The concept of* context* in Scone provides the perfect abstraction to hold episodes or situations. The way Scone handles contexts is also essential to enable episodic reasoning, since it implements a lightweight approach that barely overloads the system response as contexts are being created in the knowledge base. Moreover, the fact that only one context is active at a time provides a way of keeping inconsistent information in the same knowledge base without causing any disturbance in the data consistency.

## 3. Leveraging Common Sense

The development of the field of Artificial Intelligence has been led by the will of building computational intelligent systems. This task has turned out to be a very difficult one, and, despite the fact that computing systems have been improving their intelligent skills, the lack of common sense that they suffer from has prevented them from becoming truly intelligent. In words of Minsky [18] “*some programs can beat people at chess. Others can diagnose heart attacks. Yet others can recognize pictures of faces, assemble cars in factories, or even pilot ships and planes. But no machine yet can make a bed, or read a book, or babysit.*” In his 1968 paper [37], McCarthy proposes an approach with which to build a program with the capability to solve problems in the form of an* advice taker*. In order to do so, McCarthy reckons that such an attempt should be founded in the knowledge of the logical consequences of anything that could be told, as well as the knowledge that precedes it. In this work, McCarthy postulates that “*a program has common sense if it automatically deduces from itself a sufficiently wide class of immediate consequences of anything it is told and what it already knows.*”

For Lenat et al. [38], “common sense is the sort of knowledge that an encyclopedia would assume the reader knew without being told (e.g., an object can't be in two places at once).” Minsky [18] uses the term with regard to the things that we expect other people to know, those things labeled as obvious. In this sense, the feature that distinguishes people from computers, regarding cognitive and understanding capabilities, is the vast amount of knowledge they hold as well as their associated mechanisms that support an effective use of such knowledge.

Replicating human intelligence is therefore a task that requires an extremely large amount of knowledge. However, it is neither expert nor specific knowledge that needs to be improved in these systems. On the contrary, the focus should be placed at everyday knowledge known as common sense. In this sense, the working hypothesis motivating this work was that video-based human action recognition could be enhanced with common-sense knowledge in order to enable episodic reasoning to overcome the occurrence of nonsensical errors.

Two main difficulties are found in demonstrating this working hypothesis: on the one hand, to date, computer vision systems are not yet capable of recognizing whichever human action performed in video sequences recorded from real scenarios [1]; and, on the other hand, collecting the vast amount of common-sense knowledge held by humans is far from being a feasible task. Note that Cyc [39] has been gathering common-sense knowledge for over 25 years and it is still working on it. It is therefore necessary to make some simplifications to the original problem: human actions that are to be recognized have to be limited to a given set and human common-sense knowledge has to be reduced to an incomplete set. So, in this sense, the conclusions drawn from this incomplete set of common-sense knowledge can be directly extrapolated to the complete one.

It can be tempting to think that hand-crafted representation of expert knowledge can, at some point, replace the role of common-sense knowledge. In fact, the following quotation, extracted from [39], discusses this issue.

“It is often difficult to make a convincing case for having a consensus reality knowledge base, because whenever one cites a particular piece of common sense that would be needed in a situation, it's easy to dismiss it and say “well, we would have put that into our expert system as just one more (premise on a) rule.” For instance, in diagnosing a sick twenty-year-old coal miner, the program is told that he has been working in coal mines for 22 years (the typist accidentally hit two 2s instead of just one). Common sense tells us to question the idea of someone working in a coal mine since age-2. Yes, if this sort of error had been foreseen, the expert system could of course question it also. The argument is, however, that we could keep coming up with instance after instance where some additional piece of common sense knowledge would be needed in order to avoid falling into an inhumanly silly mistake.”

Obviously, a more careful representation of information could take into consideration that the age of a person cannot be a bigger number than the number of years the same person has been working in coal mines. Using the same context that concerns us here, it could be stated that, in order to throw something overhead, the person has to previously pick up the object that is about to throw away. However, the work presented here is more concerned with describing the knowledge that would allow the system to achieve that same conclusion on its own, rather than with providing these matching condition rules. The counterpart is that the amount of information required to do so is huge.

For that reason, the approach followed by this work consists in minimizing the common-sense knowledge involved in the considered scenario by constraining the context in which actors perform. However, it is essential to highlight that these constrains should not be equated to the approach followed by expert systems.

## 4. A Semantic Model for Human Action Recognition

There is a set of relevant concepts that characterize the process of episodic reasoning for human action recognition, independent of whether it is carried out computationally or by a person. Essentially, there is a context in which a person, typically referred to as an actor, performs a set of temporal actions, each of which is intended to a specific end. In this sense, a video-based human action recognition system only requires a concrete set of entities to model the problem domain. These are the actor who appears in the scene, the context in which the scene is framed, the actions he/she performs, the beliefs and expectations the system holds about what the actor is doing and what he/she is doing next, and finally the estimation in which all these beliefs are considered. These concepts and their relationships, expressed in a semantic model, should suffice to formally model the knowledge involved in video-based human action recognition, as empirically demonstrated in this paper.

The semantic model also provides a set of syntactic rules with their associated meaning which allows describing the knowledge involved in any episode of human action recognition. This knowledge is, in practice, reduced to a set of propositional statements written in terms of instances of these concepts and their relationships.

Finally, the need for information standardization in distributed systems also supports the demand for a semantic model. When more than one system or module interoperates to perform an operation, there exists an information exchange that should be supported on some sort of agreement that states how such information can be correctly processed and understood.

However, despite the importance of counting on a semantic model for human action recognition, a complete review of the state of the art has brought into light that this aspect has been totally overlooked. The fact that most solutions focus on the proposal of new algorithms, methodologies, or signal processing approaches is probably the reason why the knowledge management aspect of the problem has not been exploited. On the contrary, this topic has been thoroughly studied by philosophers [40–42].

Among existing theories about actions, this work implements the theory proposed by Donald Davidson in “*Theory about actions, reasons, and causes*” [40]. According to the Davidsonian view about the nature of actions, every human action is rational because the explanation of that action involves a judgment of some sort. In other words, what this theory states is that every action is motivated by an* intention*, in the broad sense of the word. So, the link between the action and the reason that explains it is what Davidson refers to as the* rationalization*.

The most relevant conclusion of this theory is that the reason that motivates an action also rationalizes it. This fact has very relevant implications to this work because it supports not only the computational formal model for human actions proposed here but also the validation of the working hypothesis motivating this work.

**Figure 1:**
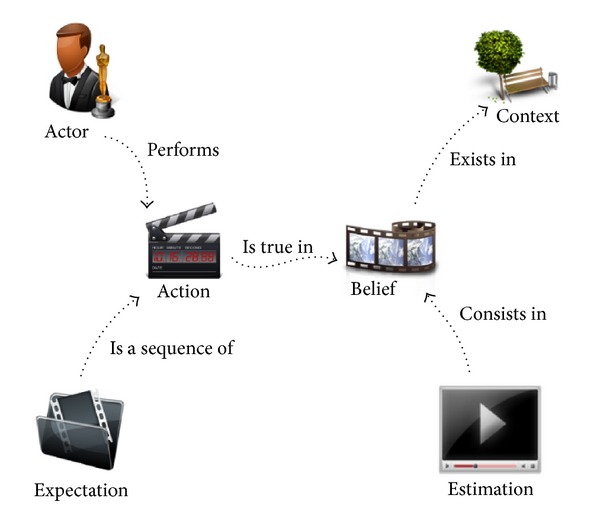
A semantic model for video-based human action recognition.

Figure 1 depicts the set of concepts and relationships that comprises a semantic model for human action recognition. Apart from the concept of action and actor, some other relevant entities require their semantics to be modeled. It is obvious that human action recognition cannot conceive existence without considering the context in which actions are being performed.

The simplicity of this model is in reducing human action nature to those concepts that cannot be avoided. This semantic model can be used to model the domain knowledge, independent of the environment in which they are being considered. Moreover, this simplicity eases the process of translating the formal model into a concrete programming language implementation of the semantic model. The following definitions state the foundation of the proposed semantic model.

**Definition 1**

*A Context* is the set *C* composed of statements which, when used together, describe knowledge about the general world or a specific belief. There may be multiple contexts describing each of the different views or beliefs of the world. The meaning or truth value of a statement is a function of the context in which it is considered.

Let us define the function meaning : *S*, *C* → *M*, where *S* is the set of statements describing the world, *C* is the set of possible contexts, and *M* is the set of possible meanings. Meaning  (*s*, *c*) returns the meaning or truth value of the statement *s* in the context *c*. This can be formally stated as

(1) $$\begin{array}{c} \exists m\in M\;\;\forall c_{i}\in C\;\;\forall s_{i}\in S:m=\text{meaning}\left(s_{i},c_{i}\right)⟺s_{i}\subseteq c_{i}. \end{array}$$

The meaning or truth value of a given statement depends on the contexts in which it has been declared.

**Definition 2**

*An Action* is the set *A* of individual actions that have been described from the perspective of their relation to the primary reason that rationalizes them. The function *AG* : *A* → *G*, such that *A* is the set of possible actions, *G* is the set of possible actors, and the function *AG* returns the actor performing the given action. Furthermore, the function *PR* : *G*, *A* → *R*, such that *R* is the set of possible reasons motivating a specific action, returns the primary reason for an actor performing an action in seeking specific results. Finally, the function *PA* : *G* → *A* returns the actions performed by an actor. Consider

(2) $$\begin{array}{c} \exists g\in G\;\;\forall a_{i}\in A:\left(AG\left(a_{i}\right)\wedge PR\left(g,a_{i}\right)\right)⟺PA\left(g\right)=a_{i}. \end{array}$$

Therefore, every action is performed by an actor if and only if there exists an actor with a primary reason to perform that action.

**Definition 3**

*An Actor* is the set *G* of individual actors who perform actions. The function *attitude* : *G* → *T* returns the set *T* of proattitudes held by an actor that support the reasons to perform certain actions. Moreover, the function *PF* : *G*, *S* → *A*, such that *S* is the subset of statements describing actions performed by actors. The function *ST* : *G*, *A* → *S* returns a statement describing that a specific actor performs a specific action at that exact time instant. Consider

(3) $$\begin{array}{c} \exists g\in G\;\;\forall a_{i}\in A\;\;\forall s_{i}\in S:PF\left(g,s_{i}\right)=a_{i}⟺s_{i}=ST\left(g,a\right). \end{array}$$

Every action performed by an actor is described by means of a time-stamped statement. Consider

(4) $$\begin{array}{c} \exists g\in G\;\;\forall a_{i}\in A\;\;\exists r\in R:PR\left(g,a_{i}\right)=r⟺r\in \text{attitudes}\left(g\right). \end{array}$$

The definition of actor therefore implies that, for every action performed by that actor and motivated by a specific primary reason, the set of proattitudes supporting the actor behavior includes that specific primary reason.

**Definition 4**

*A Belief* is the ordered set *B* of individual beliefs comprised of a temporal sequence of statements describing actions performed by actors. The function *BF* : *B* → *S* returns the sequence of action statements considered in a specific belief. Consider

(5) $$\begin{array}{c} \forall a_{i}\in A\;\;\forall g_{i}\in G\;\;\forall s_{i}\in S:ST\left(g_{i},a_{i}\right)=s_{i}⟺s_{i}\subseteq BF\left(b_{i}\right). \end{array}$$

Every statement describing the fact that an action has been performed by an actor is part of a belief.

As it has been already mentioned, the set *B* is an ordered set of individual beliefs. The order is a direct consequence of the belief grade associated with each individual belief. The more a specific belief is considered to be the real sequence of actions taking place, the higher order it has in the ordered set. The belief located at the top of the ordered sequence of beliefs is referred to as* main belief*. Consider

(6) $$\begin{array}{c} \exists mb\in B:\forall b_{i}\in B\;|\;mb>b_{i}. \end{array}$$

Finally, beliefs are not considered in isolation but as part of a more general entity called estimation. The function *BF* : *E* → *B* returns the ordered sequence of beliefs that comprise a specific estimation of a video-based analysis of human action recognition. Consider

(7) $$\begin{array}{c} \forall b_{i}\in B\;\exists e\in E:bi\subseteq BF\left(e\right). \end{array}$$

**Definition 5**

*An Expectation* is the set *X* of individual expectations; each of them contains an ordered sequence of actions that are normally referred to as activity. The function *EX* : *X* → *A* returns the ordered set of actions composing a specific expectation. Consider

(8) $$\begin{array}{c} \exists x\in X\;\exists a_{1},a_{2},\ldots a_{n}\in A:n=\left|x\right|,\\ EX\left(x\right)=\left(a_{1},a_{2},\ldots ,a_{n}\right),\\ \exists a\in A\;\exists x\in X:a\subseteq x⟺a\subseteq EX\left(x\right). \end{array}$$

Function *RA* : *X*, *A* → *A* returns the remaining ordered set of actions that follow up a specific ordered set:

(9) ∃x∈X ∃a1,a2,…,am,…,an∈A  ∃n,m∈R ∣ m<n:RA(a1,a2,…,am) =(…,an)⟺(a1,a2,…am)⊆EX(x).

**Definition 6**

*An Estimation* is the set *E* of individual estimations for each human action recognition process performed during a video sequence. An estimation consists in an ordered set of beliefs, in which the main belief is the one output by the recognition process. The function *GE* : *E* → *B* returns the ordered set of beliefs that compose that estimation. Additionally, function *MB* : *E* → *B* returns the main belief of a specific estimation. Consider

(10) ∃e∈E ∃b1,b2,…,bn∈B: MB(e)=b1⟺GE(e)=(b1,b2,…,bn).

## 5. System Implementation

**Figure 2:**
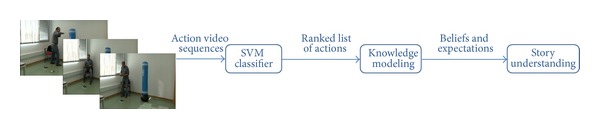
Stages involved in the proposed solution for human action recognition.

The ultimate goal of this work is to demonstrate that combining video recognition tools with episodic reasoning is the most compelling approach for human action recognition. The motivation is therefore to support the recognition process not only in video features analysis but also in the knowledge about human behavior and how the world works. In this endeavor, several stages can be identified in the proposed solution as depicted in Figure 2. The first step consists in an initial classification of actions based on visual body posture analysis. This initial classification is then provided, as input, to the knowledge-based system in charge of rationalizing the recognized actions. However, rather than proposing just one action, the computer vision system returns a list of actions whose order depends on their associated probabilities. The first action in the ordered set is the most probable one, although it does not necessarily mean that this is the correct one. For that reason, it is more sensible to consider the set of most probable actions rather than taking for granted that the most probable action, the first in the ranked list, is the correct one. This approach exploits the fact that, although the first action is not the correct one, in most cases, the groundtruth action is present in the list of the five most probable actions. Hopefully, if actors are really behaving in a rational manner, that is, performing actions motivated by reasons, and also the groundtruth action is present in that list, then we expect the reasoning system to be able to identify the correct or groundtruth action even when it has not been returned in first position. The third stage basically seeks for the motivations that might be behind each of these actions. This information supports the reasoning system in deciding which action better complies with actions believed to have been previously performed in the same video sequence.

A prototype system, going through these three stages, has been built in order to turn the working hypothesis into a real implementation that could be run and empirically evaluated. This section describes the technological decisions, grouping them into three major areas, as known, computer vision analysis, knowledge management, and common-sense reasoning.

### 5.1. Computer Vision Module for Human Action Recognition

The first stage of our system consists in generating initial action estimations by applying machine learning. Then, these estimates are passed to the knowledge-based system for further reasoning. Given a video sequence, the computer vision system, trained to recognize a given set of actions, returns an ordered sequence of actions which best describes the video according to the computer vision capability. Although each of those actions has been assessed by the system as the most probable, alternative actions may still be likely. As a consequence, in addition to the ordered sequence of actions, alternative actions with high probabilities are also provided to the knowledge-based system.

Among the different machine learning techniques that can be applied to perform human action recognition [43, 44], the Bag of Words (BoW) framework [45, 46] is particularly suitable. BoW has been proved [47–49] as one of the most accurate methods for action recognition, able to perform on a large variety of different scenarios with a low computational cost. Contrary to other classification techniques, it does not require any additional segmentation algorithm, which simplifies significantly the computer vision task and makes possible working directly on video data. Consequently, BoW methodology was chosen as the base of our computer vision module for action recognition.

Similar to most machine learning techniques, BoW relies on a training phase to learn the discriminative features and the classifiers that allow a correct recognition. Therefore, our BoW training stage consists of, firstly, producing a codebook of feature descriptors, secondly, generating a descriptor for each action video available in the training set, and, finally, training a classifier with those video descriptors. The pipeline starts by extracting salient feature points in each labeled video belonging to the training set. To ensure discriminative features, a well-known detector, Harris3D [50, 51], is applied. Once feature points are extracted from all training videos, a clustering algorithm [52] is used to group and quantize the salient point descriptors and to generate a codebook, or dictionary, which provides the vocabulary in which data will be described. Finally, each video of the training set is described in terms of the new word descriptors and used as input to train a cascade of linear Support Vector Machine (SVM) classifiers. In this way, the SVM classifiers, one per action, learn the optimal hyperplane that separate best the different actions.

During the action classification phase, actions performed in the video of interest are recognized by applying a similar procedure. Salient feature points are first detected using the same Harris3D algorithm. Then, the features are quantized using the learned codebook in order to generate a video descriptor. As final step, the descriptor is fed into each SVM classifier, which allows quantifying the similarity between the new sequence and each trained action type. As a result, an ordered list of action labels is generated according to their fit.

### 5.2. Knowledge and Semantic Model

The capability of reasoning about knowledge has become an essential feature of any system intended to intelligently behave. However, some important questions arise in relation to that knowledge: What does the system need to know in order to understand the ongoing situation? How sure the system can be about its interpretation? Whenever a conflict arises between the computer vision estimation and the knowledge-based one, which one should be considered as more reliable?

These and similar questions are formally and theoretically addressed from the knowledge model perspective. The implementation of that model is, however, not a trivial issue and several concerns need to be considered first. Selection of the most appropriate implementation technology to comply with the model requirements is one of these issues, as well as sharing the model to all modules involved in the proposed distributed architecture. This last requirement therefore imposes the constraint of being compatible with the rest of the architectural module technologies.

Regarding the first issue, ontologies, specially those written in OWL Language [53], are one of the most extended approaches to implement knowledge models. However, there are several reasons arguing against their suitability for the purpose that concerns us here. Firstly, the computer vision system returns an ordered list of actions for each single action performed in the video sequence. Although only one of those actions is selected to be part of the main belief, it is necessary to keep a record of all* discarded* actions just in case later hints suggest that a previously selected action was not correct, in which case the estimation needs to be revised to propose a different one.

The need to keep track of uncertain actions implies that* a priori* inconsistent knowledge should be asserted to the knowledge base. Inconsistency issues arise when propositional statements describe the actor performing different actions at the same time instant. These same time-instant actions correspond to each of the actions returned by the computer vision module. For example, if two of the actions of the set are sitting down and getting up from a chair, two propositional statements stating these facts should be asserted to the knowledge base. Obviously, this situation would lead to an inconsistent situation since both actions cannot be performed at the same time.

Philosophers [41, 42] have suggested a theory to tackle the problem of how to deal with inconsistent knowledge. This theory has been extrapolated to computing and, according to Hobbs and Moore [54], instead of talking about the propositions that are true in a given context—or belief, using the terminology proposed here—one should rather talk about what states of affairs are compatible with what is already known. These states of affairs are referred to by philosophers as* possible worlds* [55]. The possible worlds theory basically consists in creating different worlds—once again we can talk about beliefs—each of which comprises the propositional knowledge verified to be consistent.

This leads to isolating inconsistent facts in different knowledge islands, referred to here as* beliefs*. Consistency issues can therefore be avoided by considering true only the knowledge described under the active belief. In this sense, each of the actions returned by the computer vision module, instead of being asserted to the general knowledge base, is being individually asserted to a different belief. This approach assures that the general knowledge base is consistent, as well as each of the different beliefs created in each estimation process.

Implementing the possible world theory to describe the propositional knowledge comprised of each belief has several advantages: (a) standard automatic deduction and inference techniques can be applied; (b) it assures knowledge-based consistency; (c) and more importantly uncertain information does not need to be discarded.

Unfortunately, ontologies do not yet enable the representation of possible worlds due to the impossibility of deactivating some parts of the ontology while keeping the rest active. This mechanism is not supported by neither ontologies nor the existing approaches to manage them, such as Protege [56]. On the contrary, Scone represents an excellent option to deal with possible worlds by means of its* multiple-context* mechanism [4, 57]. Every world or every belief can be described in a particular context, and only one context at a time is active. Only the knowledge described in the active context is considered, therefore avoiding inconsistency issues among statements asserted to different contexts.

Not being able to deal with* a priori* inconsistent knowledge is not the only reason why ontologies cannot be used for the proposed architecture. In addition, although several frameworks claim to support ontology reasoning [58–60], they are actually only performing consistency checking operations. In this regard, Scone provides powerful mechanisms to support real reasoning tasks. The* marker-passing* mechanism [36] that it implements provides an extremely efficient way of performing inference, deduction, or reasoning by default operations.

In summary, the use of ontologies is unsuitable for the purposes described here, whereas Scone, through the multiple-context and marker-passing mechanisms, represents an excellent option for fulfilling the requirements of the proposed semantic model and the knowledge management demands.

**Figure 3:**
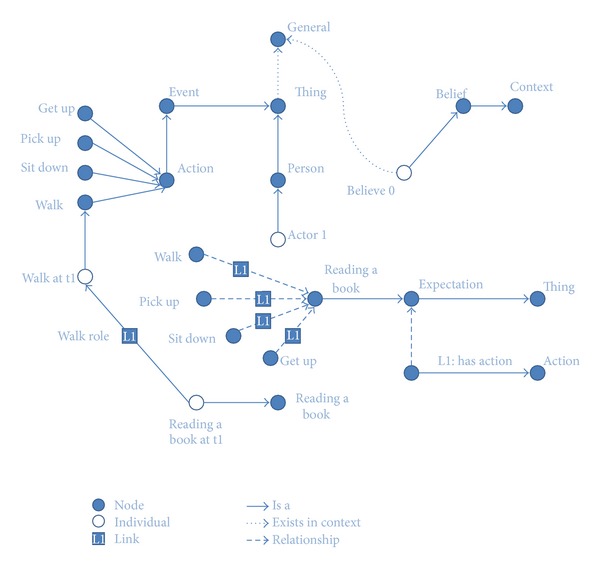
Knowledge modeled using Scone.

Once the election of the Scone knowledge-based system has been justified, the next matter to be tackled is the implementation of the proposed knowledge model using the Scone language syntax.  Figure 3 depicts implementation using the Scone terminology.

The Scone language is a dialect of Lisp, in which new concepts and relationships can be easily created to represent all the elements of the semantic model.  Sconecode 1 shows how the actor concept is created as a specialization of one person, therefore inheriting all the properties and relationships of a person. Still, the actor concept is a high level entity. This entity can be made concrete by means of the* individual* abstraction. Whenever an individual of a certain type is declared, an instance of that type is created.

Finally, this module does not only consider the semantic model knowledge or the knowledge describing how the world works, also known as common-sense knowledge, but also it does count on domain specific knowledge. Domain specific knowledge can be also referred to as context knowledge. However, for simplicity purposes, we will refer to that as domain specific knowledge (DSK) to avoid confusions with the word* context* that was previously used to describe the mechanism implemented by Scone to support the possible world theory. DSK consists in the propositional knowledge that describes the environment in which actions are being performed. This information turns out to be essential for meaning disambiguation purposes. DSK is also described using the Scone language and asserted to the Scone knowledge-based system, in the general context, inherited by every belief context.

Sconecode 2 shows some of the propositional statements describing the DSK of the particular scenario.

This code sample shows how basic information about the environment is described under the proposed framework. This code represents the description of a test room, in which there is an entrance or doorway, as an example of domain specific knowledge (DSK). The common-sense knowledge—also referred to as world knowledge or WK—already holds propositional statements stating that entering a room is an action that consists in crossing through a doorway to enter an enclosed space of a building. In addition, there is also a chair in the room—example of DSK—which is a type of sitting surface—example of DSK. In the same way, the other elements present in the test room are described following similar rules.

### 5.3. Common-Sense Reasoning Module

This section describes the world knowledge functioning and the way people behavior can be heuristically used to recognize human actions. More specifically, this section intends to replicate the foundations for human's ability to recognize actions when angles or video quality is quite poor, for example, and visual information is scarce or insufficient to support the recognition task.

As stated in Section 5.2, the proposed framework for knowledge modeling is based on the possible world theory. This theory is the enabling key for modeling cognitive mental models such as* beliefs*, through the use of the* world* abstraction. Section 5.2 also states that the possible world theory is implemented in Scone by means of the multiple-context mechanism. This subsection is now concerned with how to implement the reasoning routines exploiting the semantics implicit in the possible world theory.

**Figure 4:**
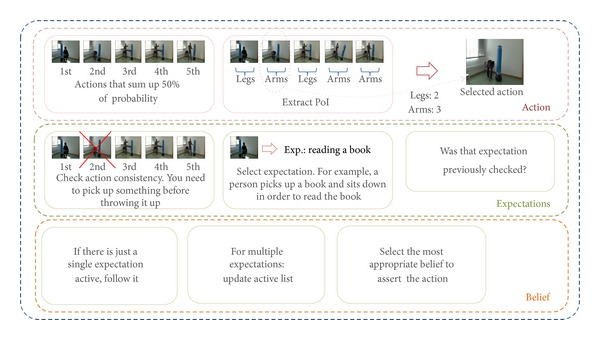
High level stages involved in the reasoning process.

Figure 4 depicts a high level description of the proposed reasoning routine. According to the entities involved in the different routine stages, three different levels can be identified. The first level deals with the action set returned by the computer vision system. Every human action can be characterized by the body part that mainly intervenes in accomplishing that action. For example, the punching action mainly involves fists, as part of arms, whereas the kicking one involves legs. The action set is therefore analyzed in order to determine the prevailing body parts. The body part, or so-called here point of interest (PoI), that more frequently appears in the action set is used to reorder the same action set so that first actions are those involving the PoI, delegating others to the bottom of the list. Given that kicking, punching, waving, and scratching head are examples of an action set, the arm is the body part that appears more often. This means that the first action, or the most probable action, is not kicking as it was originally estimated, but the next most probable one involving the arm, which is, in this case, the punching action. Then, the reordered list of actions is checked for inconsistency issues. Consistency checking consists in determining whether the action requirements are fulfilled. Those actions whose requirements are not fulfilled are discarded.

The second level considers the case where an action is part of a composite or activity, here referred to as expectation. When an expectation is active, it can be assumed that the actor is engaged in accomplishing that sequential set of actions. For that reason, given the actions already performed by the actor, it is possible to know the actions that are expected next.

It might be possible that more than one expectation is active at a time. In that case, the system should keep track of them which will lead to not forcing any belief of the actions to come. Alternatively, if the active expectation is unique, the next action to come is sought in the ordered action set and afterward asserted to the main belief. If the expected action was not in the list, the action will be forced to the main belief. Remaining actions are asserted to the following beliefs.

**Figure 5:**
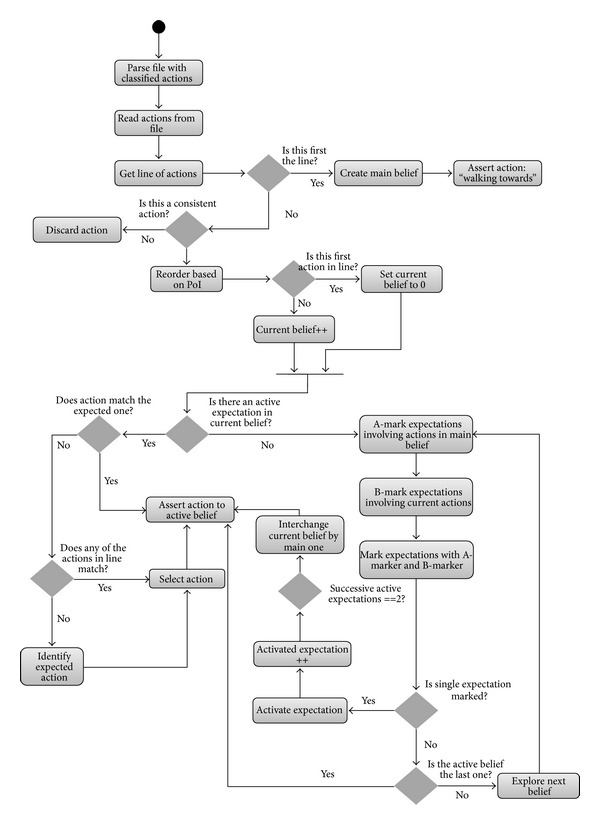
Activity diagram for reasoning about human actions.

**Figure 6:**
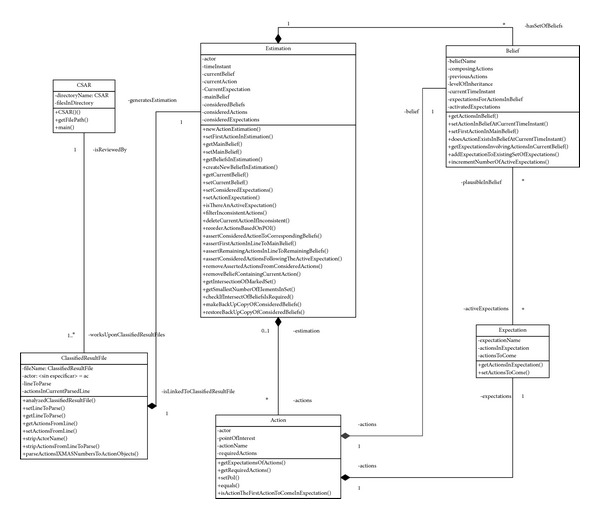
Class diagram for the common-sense reasoning module.

Figure 5 depicts the activity diagram of the previously described process, whereas Figure 6 shows the class diagram for the implementation routine.

Finally, going one step further in the level of details used to describe the reasoning routine, Algorithm 1 shows its implementation using pseudocode. Whereas the focus of this work is on describing the proposed knowledge framework, the work in [5] provides a thorough description of the algorithmic aspects.

This condensed version of the proposed reasoning mechanisms essentially analyzes each of the five actions provided by the computer vision system. These actions are evaluated in the context in which they are being considered, referred to as DSK and, in the context of general knowledge, referred to as WK. If actions are consistent with this information, they are studied, in order to determine whether any of them is part of an activity. If the analysis of the current and past actionss brings into light that there is a unique activity taking place, then the activity is said to be active. Active activities—also referred to here as expectations—drive the system in its decision of which actions to assert in each of the parallel considered beliefs.

## 6. Experimental Validation

Following descriptions of both the theoretical and implementational aspects of the working hypothesis, this section is devoted to discussing validation issues. It is important to recall that the working hypothesis motivating this work was that the combination of common-sense knowledge, reasoning capabilities, and video-based action classification could improve human action recognition of each of the parts in isolation. This is done by removing common-senseless mistakes which introduce noise into the recognition process.

The main axiomatic fact supporting this working hypothesis is directly drawn from the nature of human agency or human behavior. Based on the Davidsonian view of human agency [40], actions are always motivated by a primary reason. Supported in this philosophical premise, the reason that motivates actions also rationalizes them.

This section aims at demonstrating the correctness of the working hypothesis. Since the proposed solution is tackled from taking advantage of different perspectives, that is, the computer vision and human cognition, each of them has to be validated.

In this sense, the followed approach consists in comparing the accuracy of a common-sense based computer vision system with one without reasoning capabilities. Moreover, the human cognitive perspective is validated by comparing the system with the most characteristic example of cognitive subjects: people. This was achieved by asking people to perform the same recognition tasks performed by the system and under the same circumstances. Comparison between the system and people performances allows assessing the level of “*commonsensicality*” held by the proposed system.

### 6.1. Accuracy Assessment

Traditionally, the best way for assessing the performance of computer vision system for human action recognition is by training and testing the proposed system with one of the publicly available datasets. These open and public datasets are therefore the most suitable benchmarks for evaluating and comparing proposed solutions with existing approaches.

Despite the relevant number of existing datasets, none of them fulfill the initial requirements of this work, which include encapsulating the complexity of real life applications with a significant number of complex activities. The lack of rationality with which the actions of these datasets are performed made them unsuitable for the purposes of this work. Indeed, the proposed solution is based on the premise that actions had to be performed for a reason in order to be rational. Unfortunately, existing datasets consist in video sequences in which actors are told what to do in a contextless scenario. Actions have to be part of a comprehensive story, so that performed actions make sense with regard to the aims or the reasons that motivate actors to behave like they do.

Two main premises support the creation of the new dataset: first, rule-based strategies are to be avoided; and, second, directions given to actors are kept to a minimum. These two premises can be satisfied by creating the appropriate atmosphere that makes actors prone to perform certain actions but allowing them, at the same time, to behave in a rational manner.

If the reason that motivates an action also rationalizes it, and, consequently, if motivations could be heuristically driven and restricted, actions would also be limited to those matching the available motivations. In other words, if we want a person to perform a certain action all what has to be done is to motivate that person to do so. If that motivation is subtle enough, this implication can be used to demonstrate that common-sense capabilities enhance the performance of a computer vision system. Recall that it is necessary to limit the set of actions performed in a scene because, on the one hand, the available common-sense knowledge-based system is incomplete and, on the other hand, the computer vision system is only capable of recognizing a small set of actions. It has to be highlighted that actors should not be instructed to perform specific actions, because, by doing so, the rationality explaining the action would have been contaminated. On the contrary, by creating the appropriate atmosphere to motivate certain actions, we are creating a real scenario, in which actors act driven by intentions, while at the same time assuring that the scenario remains in the boundaries of the set of actions and knowledge known by the system.

The limited number of actions that can be recognized by computer vision systems justifies the need for a set-up scenario. There, actors are surrounded by suitable elements that encourage them to perform a predefined and expected set of actions such as punch or kick a punching-ball or read a book. The negligible probability of an actor performing those activities without the presence of interactive objects for fighting or reading makes them necessary for capturing the actions of interest.

The proposed scenario consists in a waiting room in which several objects have been strategically placed. Objects such as a punching-ball, a chair, or a book are motivating people behavior, for example, to play with the punching-ball which should lead them to perform the kicking and punching actions or to sit down on the chair.

Eleven sequences were recorded in which actors were just told to remain in the room for a period of time and feel free to enjoy the facilities present in the room. These eleven sequences were manually groundtruthed and segmented into actions. Afterward, these actions were fed to both, the basic computer vision system and the enhanced version in which common-sense capabilities had been leveraged.

In order to train a computer vision system capable of successfully detecting and segmenting the actions happening on this testing scenario, a suitable dataset must be chosen. This training dataset must not only comprise similar activities to the ones being promoted in our testing contextualized dataset but also fulfill a set of requirements. Thus, the training set must be able to cover a variety of camera views so that recognition is view-independent and the set should include a sufficiently large amount of instances of the actions of interest. These instances must be not only annotated but also perfectly segmented and organized to simplify training. The only suitable sets which fulfill these requirements and cover most of the activities that are promoted for our testing environment are IXMAS [43]. IXMAS is focused on standard indoor actions which allows providing quite an exhaustive description of possible actions in our limited scenario. Since it is comprised of 12 actions, performed by 12 different actors, and recorded simultaneously by 5 different cameras, it provides view independence and should offer sufficient examples to train a discriminative action classification.

Table 1 shows the average of the accuracy rates obtained for the eleven video sequences. A closer look to the accuracy rates obtained by actor shows that, in the best case scenarios, accuracy rates reach a 75% of positive recognition for the enhanced system.

Table 2 presents the accuracy rates obtained by both systems, the basic and the common-sense enhanced one, for each individual actor. The columns with labels 1 to 11 represent each of the 11 individual actors, each of which has been recorded in a video sequence. As it can be seen in that table, even when using the same recognition approach—basic or common-sense enhanced—accuracy rate experiments dramatic variations. Several reasons explain these values, mainly based on the rationality with which actions were being performed by each actor. However, since these aspects belong to the human cognition side, it will be more detailed and analyzed in the next subsection.

Note that results shown in Tables 1 and 2 were initially presented in [5].

### 6.2. Commonsensicality Assessment

From the cognitive perspective, this system claims to hold common-sense knowledge and reasoning capabilities complementing the computer vision system in the task of recognizing human actions rationally performed. Assessing this claim is not a trivial matter, mainly due to the fact that common-sense knowledge is neither unique nor common to everybody. On the contrary, common sense varies from one person to another due to criteria such as age, gender, education, or culture.

Measuring how commonsensical a person or a system is resembles the problem of measuring human intelligence. Traditionally, intelligence quotients have been used to determine and compare intelligence levels among humans. These quotients are obtained from performance of subjects in intelligence tests. The same approach is therefore followed here to measure the commonsensical level of the system in comparison to humans. Rather than resorting to complex and philosophical questionnaires about common-sense knowledge, the proposed approach consists in presenting humans to the same situations analyzed by the system and comparing their answers. In the aforementioned intelligence tests, intelligence is treated as though it was unique and common to every human being, with the disadvantages involved in this simplification. However, if results are interpreted within the boundaries of these simplifications, this type of test can be very useful. In other words, intelligence test cannot be considered to be the silver bullet for determining how intelligent a person is, but, if they are correctly addressed, they can certainly bring into light very relevant information about certain aspects of human intelligence. This fact is highlighted here in order to make sure that results retrieved from the proposed questionnaires are not misused or misinterpreted, and they are considered within the boundaries in which they were conceived.

Obviously, if humans were provided with video sequences they would easily figure out the actions being performed. Moreover, the performance of the vision system has been already stated in the previous section. For both reasons, subjects will be presented with the same information provided as input to the common-sense reasoning system: the set of the five most probable actions returned by the computer vision system. Based on that action set and the description about the scenario in which actions are being performed, humans have to determine the course of actions taking place. In order to allow a fair comparison, the people completing the questionnaire have also been provided with a full description of the environment and the actions actors can perform. The questionnaire has been elaborated allowing people to change previous estimations based on information from following actions; in the same way the common-sense reasoning system interchanges beliefs whenever a lower-priority belief starts gaining credit, due to the sequential activation expectations.

Since humans, unlike machines, easily get fatigued when engaged in tedious and repetitive tasks, the questionnaire should be therefore compiled to mitigate the impact of tiredness in the obtained result. The proposed approach consists in focusing on the two most extreme cases, that is, those in which the recognition accuracy rates obtained by the system are the highest and the lowest. Looking back to Table 2, in which the system performance is compared with a computer vision system, it can be noticed that actors 4 and 10 are, respectively, those in which the highest and lowest accuracy rates are achieved.

Results show how, despite some variations, the majority of the people tend to identify certain actions with the same estimation. This suggests that the mental process followed to reason about human actions is quite similar among people, independent of nationalities, education, or age. However, there are always subjects in the groups who disagree. This probably means that they are following a different reasoning course.

Independent of the mental process followed by questioned people, it has to be highlighted that, when compared with the system under test, they do not outperform the system accuracy rate. In fact, people even degrade performance of the computer vision system. This fact can therefore be used to demonstrate that the proposed system works, at least, as well as a representative sample of people. This fact also indicates that the common-sense approach used by the system better suits the characteristic of the problem, if compared with the mechanisms employed by the questioned people. Probably, people are resorting to more complex mechanisms such as past experiences, for example, that are encouraging them to ignore the recommendations of the computer vision system. It is also probable that those who have had previous experiences with computer vision or intelligent systems better understand the mechanisms of these systems. Consequently, these people provide estimations more similar to the ones provided by the system. This is indeed one of the boundaries constraining the importance that should be given to the questionnaire results.

It is also worth mentioning that actor 4, the one behaving in a more rational manner, obtains a higher recognition rate than actor 10, the one behaving more erratically. Questionnaire results demonstrate that, as expected, a rational behavior can be more easily recognized than an erratic one. In this sense, accuracy rates obtained by questioned people are always better for actor 4 than for actor 10. This is the most relevant conclusion drawn from the analysis of the questionnaire results, since it can be used to demonstrate one of the axiomatic facts motivating this work: common-sense capabilities improve recognition rates of rational behavior.

The following tables summarize the most relevant aspects of the undertaken test.  Table 3 starts by summarizing the different subjects that have participated in these tests. Thirty-seven people, from six different nationalities and various age groups, have performed the questionnaires. Additionally, Table 4 summarizes accuracy average obtained by the 37 questioned subjects. These values are compared with the ones obtained by the system proposed here. Finally, Table 5 shows the accuracy rate obtained in the recognition of each of the 12 actions composing the analyzed sequence.

## 7. Conclusions

This paper describes a system for video-based human action recognition enhanced with common-sense knowledge and reasoning capabilities. The main motivation of this work was to demonstrate that computational tasks involving some degree of human behavior understanding cannot be successfully addressed without considering some form of reasoning and contextual information. To demonstrate this axiomatic fact, a system has been built combining both strategies: computer vision and common sense.

The proposed system performs a primary recognition of actions, which is only based on image analysis capabilities. This first stage calculates the five most probable actions according to actors body postures. These actions are provided as inputs to the common-sense reasoning system. In a second stage, the common-sense reasoning model performs some reasoning tasks upon the computer vision system suggested actions. These operations are supported upon a formal model of knowledge, also proposed and formalized here.

Essentially, three conceptual abstractions are proposed in this model in order to replicate the mental process followed by humans into a computational system. The notion of action, belief, and expectation articulates the reasoning mechanisms implemented according to the Davidsonian theory of actions. In order to validate this model, a new video dataset has been proposed here, in which actions are motivated by reasons. The environment in which those video sequences are recorded has been carefully designed to provide actors with the reasons to perform the actions known by the computer vision system. This contribution is validated by the construction of the prototype, therefore verifying that the proposed semantic model complies with knowledge requirements arising in supervised contexts for human action recognition.

Two more aspects need to be validated, as they are the performance of the system in terms of recognition rates and* commonsensicality*. The first aspect has been evaluated by implementing a state-of-the-art approach for vision-based human action recognition. The second aspect is evaluated by asking people to recognize human actions, based on the sole information provided by the five most probable actions. Results in both sides demonstrate that incorporating common-sense knowledge and reasoning capabilities dramatically improves recognition rates. Additionally, it can also be concluded from the questionnaire analysis that, in order for the common-sense reasoning system to show its great potential, human actions being analyzed should be part of the rational behavior of the actor. Both the common-sense reasoning system and people have failed to successfully recognize actions performed by erratic actors.

Finally, it should be highlighted that this work tackles the problem of vision-based human action recognition from a comprehensive perspective. This entitles the proposed system to be deployed in any supervised environment in which human behavior understanding is required, as in Ambient Assisted Living.

## Figures and Tables

**Algorithm 1:**
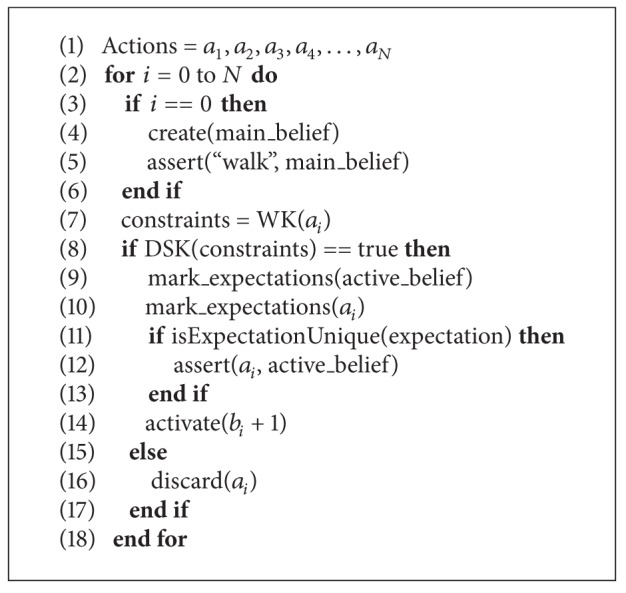
Perform estimation (actions).

**Sconecode 1:**
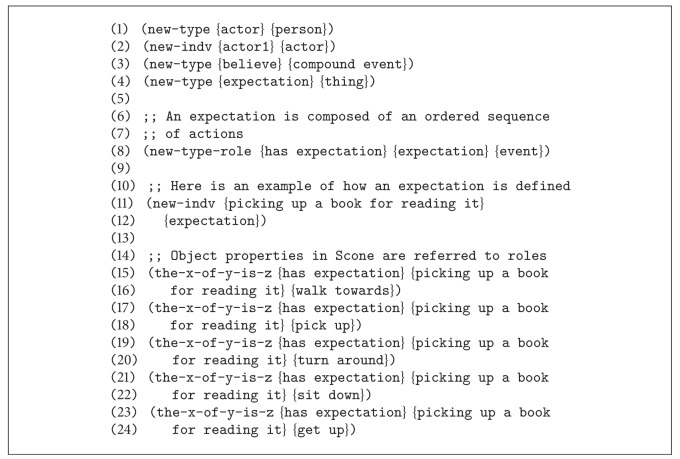


**Sconecode 2:**
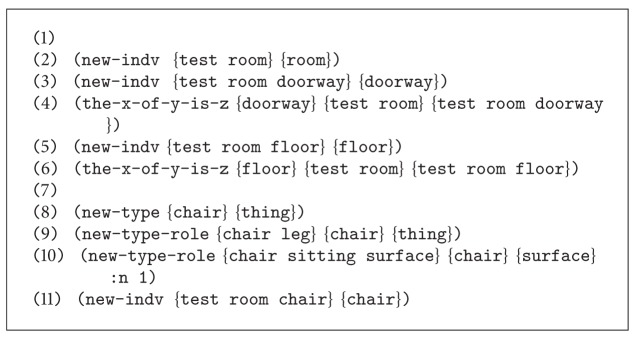


**Table 1:** Average of accuracy rates obtained by the basic and common-sense enhanced system.

Computer vision system	Accuracy rate
Basic computer vision system	29.4%
Common-sense based computer vision system	51.9%

**Table 2:** Accuracy rates for each individual actor.

CVS	1	2	3	4	5	6	7	8	9	10	11	Avg.
Basic	35.5	16.0	30.0	58.3	44.4	22.2	40.0	15.4	40.0	16.7	33.3	29.4
CS	64.5	52.0	50.0	75.0	55.6	66.7	40.0	30.8	60.0	25.0	33.3	51.9

**Table 3:** Participants information.

Gender			Age				Education			Nationality (6 in total)				
Male	Female	?	<25	25–40	>40	?	Undergrad	Postgrad	?	Spanish	Other EU	Asian	Canadian	?
34	3	—	9	11	4	13	9	15	13	16	5	2	1	13

**Table 4:** Average of accuracy rates obtained by questioned people and system.

System	Actor 4 (%)	Actor 10 (%)
Questionnaires	43.01	25.67
Reasoning system	75.0	25.0

**Table 5:** Accuracy in % obtained in recognizing each action.

Actor 4	Walk	Punch	Point	Walk	Punch	Turn	Walk	Punch	Turn	Punch	Check	Walk
	100	75.67	16.21	5.40	54.05	0	2.70	64.86	10.81	56.75	48.64	94.59

Actor 10	Walk	Kick	Turn	Walk	Punch	Walk	Scratch	Walk	Sit	Get	Wave	Walk

	97.29	0	2.70	18.91	48.64	2.70	2.70	16.21	5.40	5.40	16.21	91.89
